# DGAT inhibition at the post‐absorptive phase reduces plasma FA by increasing FA oxidation

**DOI:** 10.15252/emmm.202318209

**Published:** 2023-10-04

**Authors:** Anand Kumar Sharma, Christian Wolfrum

**Affiliations:** ^1^ Laboratory of Translational Nutrition Biology Institute of Food, Nutrition and Health, ETH Zurich Schwerzenbach Switzerland

**Keywords:** Metabolism

## Abstract

In this Correspondence, A. Sharma & C. Wolfrum report that DGAT1/2 pharmacological inhibition at post‐absorptive phase in mice leads to increased fatty acid oxidation and reduced plasma fatty acid levels, which could open new therapeutic avenues to avoid GI complications observed in clinical trials.

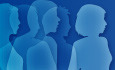

Diacylglycerol O‐acyltransferase 1 and 2 (DGAT1/DGAT2) are two enzymes that catalyze the last reaction of triglyceride synthesis (Chitraju *et al*, 2019; Sharma *et al*, 2023). In humans, DGAT1 seems to be indispensable for dietary lipid absorption (Haas *et al*, 2012). In mice, DGAT2 efficiently compensates for the loss of DGAT1 activity in adipose tissue while intestinal compensation has been a matter of speculation (Chitraju *et al*, 2019; Takemoto *et al*, 2020). Consequently, although *DGAT1* loss‐of‐function in humans causes severe diarrhea‐like symptoms (Haas *et al*, 2012), *Dgat1* knockout mice are healthy and resistant to diet‐induced obesity (Chen *et al*, 2002). Only a combined inhibition of DGAT1 and 2 causes diarrhea‐like symptoms in mice (Takemoto *et al*, 2020).

Based on the healthy metabolic phenotype of *Dgat1* knockout mice (Chen *et al*, 2002; Chen & Farese, 2005) multiple DGAT1 inhibitors to treat obesity and metabolic disorders were developed and tested in the last 10 years (DeVita & Pinto, 2013; Amin *et al*, 2023). However, due to gastrointestinal complications in humans (DeVita & Pinto, 2013; Amin *et al*, 2023), the clinical trials have faced a setback, and subsequently, DGAT1 inhibitors were given a low priority as anti‐obesity/insulin sensitizers, partly due to the notion that DGAT1i mainly acts through inhibition of intestinal absorption of dietary lipids.

We observed an absorption‐independent effect of combined DGAT inhibition in mice when administered 5 mg/kg doses of well‐characterized DGAT1 (PF‐04620110) and DGAT2 (PF‐06424439) inhibitors (D1 + 2i) 3 h after the food withdrawal to minimize the intestinal lipid absorption burden. In normal chow‐diet‐fed (NCD) lean as well as high‐fat diet‐fed (HFD‐fed) obese mice, D1 + 2i induced a striking reduction in the plasma fatty acids (Fig 1A and B). In the normal chow‐fed animals, D1 + 2i led to ~25% reduction in plasma FA levels (Fig 1B, left panel) while plasma insulin (Fig 1B, middle panel) or blood glucose levels (Fig 1B, right panel) were minimally affected. In HFD‐fed mice housed at room temperature, D1 + 2i caused > 50% reduction in the plasma FAs at a 4‐h timepoint while plasma glycerol and glucose levels were minimally affected (Fig 1C). We then asked if D1 + 2i is only effective in clearing the FAs derived from the basal lipolysis or if it could also clear excess FAs at the stimulated lipolysis states. To examine this, we exposed HFD‐fed mice to cold temperatures which led to slightly increased plasma FAs. In this case, 2‐h FA clearance was comparable to RT (Fig 1D) and reached a plateau, possibly as a mechanism to maintain FAs for other organs to use as energy sources. We then used indirect calorimetry to test the fuel preference upon D1 + 2i. A reduction in the respiratory exchange ratio (RER) suggested that after D1 + 2i treatment, mice preferentially used fatty acids as an energy source (Fig 2A and B). The difference in RER was more pronounced in cold (Fig 2B, middle panel). To prove the absorption independence of the RER phenotype, we designed a re‐feeding experiment. Mice were first fasted for 5 h, which led to a lower RER as expected. We then injected D1 + 2i (or vehicle) and food was provided thereafter. In this case, owing to reduced intestinal lipid absorption, D1 + 2i caused a higher RER than the vehicle group, suggesting that the 3‐h food removal regimen effectively dissociates the absorption‐related effects (Fig 2B, lower panel).

**Figure 1:**
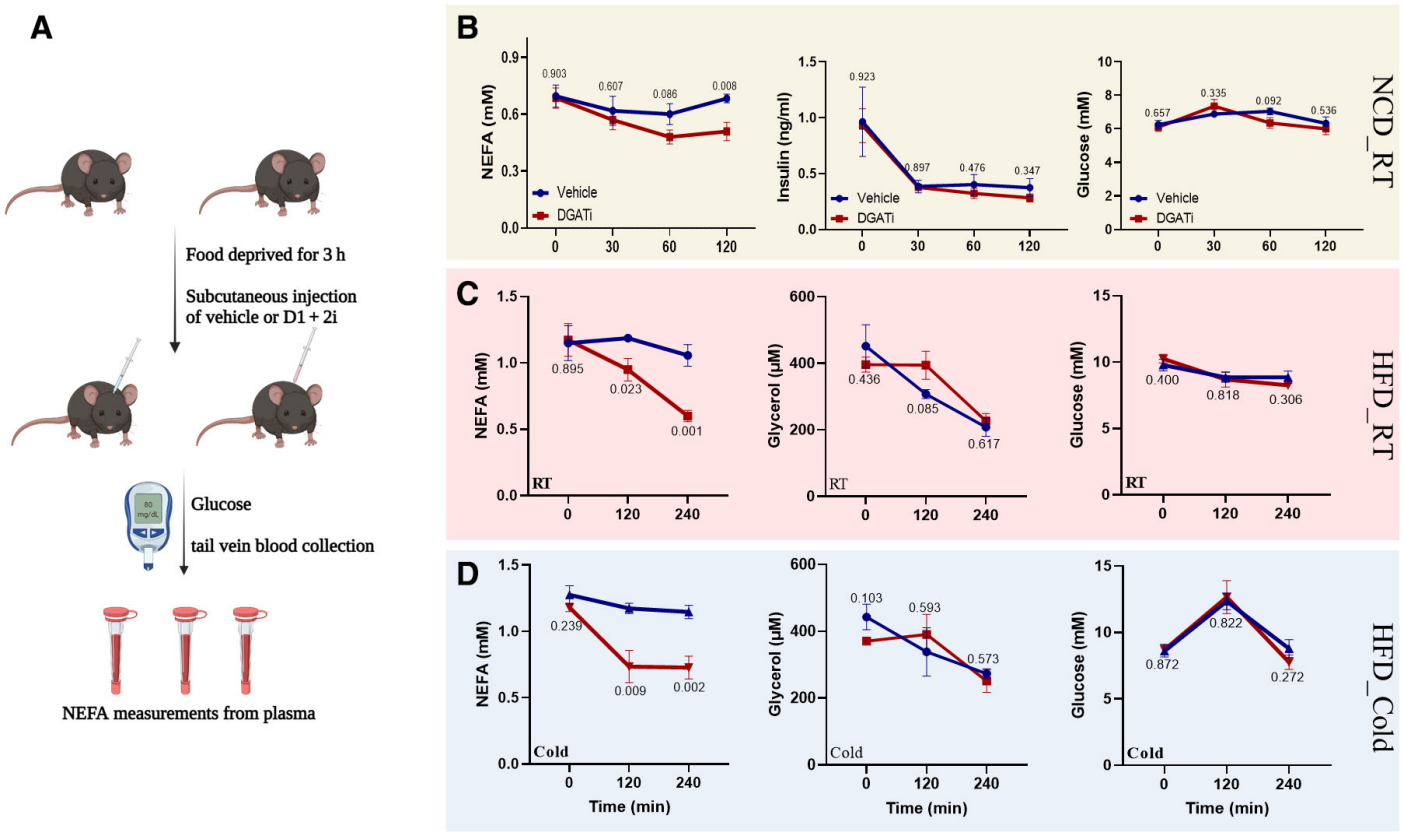
D1 + 2i reduces circulating FA levels (A) Schematic depiction of the experimental approach for plasma analysis from HFD‐fed mice. (B) D1 + 2i induced changes in plasma FA (left), plasma insulin (middle), or blood glucose levels (right) in normal chow‐fed mice. Data are presented as mean ± SEM, *n* = 7–8. (C) D1 + 2i induced changes in plasma FA (left), plasma glycerol (middle), or blood glucose levels (right) in high‐fat diet‐fed mice at room temperature. Data are presented as mean ± SEM, *n* = 5. (D) D1 + 2i induced changes in plasma FA (left), plasma glycerol (middle), or blood glucose levels (right) in high‐fat diet‐fed mice exposed to cold. Data are presented as mean ± SEM, *n* = 5. *P*‐values (mentioned in the graphs) were calculated by two‐tailed student *t*‐test at individual timepoint. The blue line represents the control group while the red lines represent the D1 + 2i group.Source data are available online for this figure.

**Figure 2:**
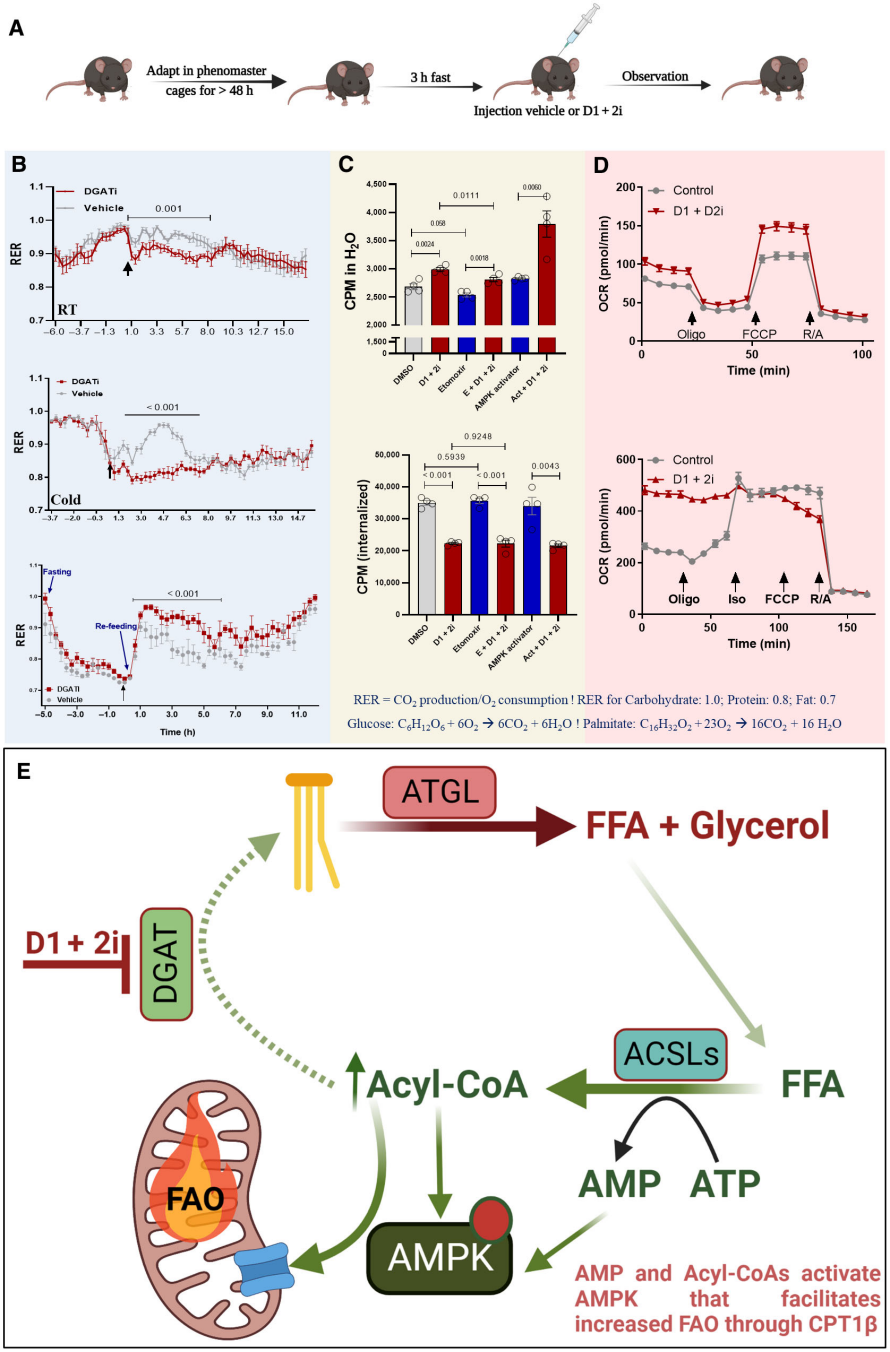
D1 + 2i promotes FAO (A) Schematic representation of the experimental setup for indirect calorimetry to assess fuel utilization. (B) Indirect calorimetry data shows a reduced respiratory exchange ratio (RER) after D1 + 2i treatment compared to the vehicle‐treated group at room temperature (upper panel), or in cold (middle panel). A fasting experiment was performed after a 4‐day adaptation at RT (lower panel). The food was removed 5 h before vehicle/drug injection. After drug administration, the mice were provided with food. Data are presented as mean ± SEM. *n* = 4–5. *P*‐values (mentioned in the graphs) by a two‐way ANOVA for the data from the selected duration. The black arrows represent the time of inhibitor injection. (C) *in vitro* FAO assay showing that D1 + 2i treated adipocytes show an increased FAO (upper panel) without affecting the cellular uptake of exogenous FA. Etomoxir slightly decreased the FAO while AMPK activator slightly increased the FAO. The FAO in D1 + 2i + etomoxir treatment was lower than in D1 + 2i alone. In contrast, the D1 + 2i and AMPK activator combination increased the FAO. Data are presented as mean ± SEM, *n* = 4. *P*‐values (mentioned in the graphs) were calculated by group‐wise comparisons by a two‐tailed student *t*‐test. (D) Seahorse extracellular flux analysis to measure the effect of D1 + 2i on the oxygen consumption rate (OCR) as a readout of cellular FAO (Sharma *et al*, 2023). HepG2 cells (upper panel, 30,000 cells per well) or *in vitro* differentiated brown adipocytes (lower panel, replated on day 5 of differentiation at the density of 10,000 cells per well) were cultured on 96‐well extracellular flux measurement culture plates. HepG2 cells were assayed the next day while adipocytes were analyzed 48 h after the replating. Cells were washed with minimal DMEM (provided with a seahorse kit) which was supplemented with glucose, pyruvate, and glutamate (Sharma *et al*, 2023). The vehicle or D1 + 2i was dissolved in seahorse mediated and treated for 1 h at 37°C. Indicated inhibitors (oligomycin, isoproterenol, FCCP, and Rotenone/antimycin) were filled in the cartridge wells, and measurement was performed on the XFe96 extracellular flux analyzer. Data are presented as mean ± SEM. *n* = 5. In both cases, D1 + 2i led to a significant increase in basal oxygen consumption (**P < 0.001). (E) A graphical abstract of the phenomenon of D1 + 2i‐induced reduction in FAs via increased FAO. In adipocytes, basal lipolysis generates FAs that are simultaneously re‐esterified to triglycerides by DGAT1 or 2. Upon D1 + 2i, this pool of FAs (activated FA = Acyl‐CoA) is diverted mitochondria for FAO. AMPK activation and downstream signaling seem to be important for this diversion (Sharma *et al*, 2023).Source data are available online for this figure.

We validated the effect of D1 + 2i on fatty acid oxidation (FAO) *in vitro* in adipocytes and found that D1 + 2i directly increased FAO (Fig 2C). We included etomoxir as the inhibitor of the FAO. We recently identified AMPK as a crucial mediator of DGAT action in adipocytes (Sharma *et al*, 2023). Moreover, it is known that Acyl‐CoA can directly activate AMPK (Pinkosky *et al*, 2020). Therefore, we tested if activation of AMPK can potentiate the FAO by D1 + 2i. The effect of D1 + 2i on FAO was diminished by etomoxir while a combination with an AMPK‐activator further increased the FAO (Fig 2C). The presence of either etomoxir or an AMPK‐activator did not affect the activity of D1 + 2i on intracellular accumulation of exogenous palmitate (Fig 2C). We next tested if cell types other than adipocytes also similarly respond to D1 + 2i. In this regard, we recently showed that an increase in oxygen consumption rate (OCR) is a hallmark of D1 + 2i‐induced channeling of FA for FAO (Sharma *et al*, 2023). We used the same approach with HepG2 cells and observed a slight increase in OCR (Fig 2D, upper panel). However, this increase was less pronounced than adipocytes (Fig 2D, lower panel), suggesting that although adipose may be the primary effector of D1 + 2i mediated reduction in FA likely through FAO, other organs (e.g., liver and muscle) may also be a secondary contributor. We propose that the D1 + 2i response is directed towards the disposal of excess FA by FAO due to the blockade of re‐esterification by D1 + 2i.

## Discussion

Taken together, these data suggest that the reduction in FAs by D1 + 2i may be a potential mechanism of insulin sensitization independent of dietary lipid absorption similar to other insulin‐sensitizing agents (Guan *et al*, 2002). Recent findings that adipose‐specific combined deletion of *Dgat1* and *Dgat2* (double knockout) imparts resistance to diet‐induced obesity, which is associated with increased energy expenditure and reduced RER further support the idea that DGATs potentially improve metabolic health independent of intestinal absorption (Chandramohan *et al*, 2023). Therefore, it will be clinically interesting to test D1 + 2i a few hours after a meal (DGAT1i alone or D1 + 2i, e.g., late at night) to minimize absorption‐related gastrointestinal side effects while imparting insulin sensitization. Moreover, considering the combinatorial effect of the AMPK activator, a combination of D1 + 2i with intermittent fasting or caloric restriction seems a promising alternative regimen.

Several aspects need further consideration for accurate data interpretation, including the extent of absorption‐independence of the phenotype. We observed a visually appreciable extent of watery diarrhea after a single dose of D1 + 2i in the HFD‐fed mice which worsened after 2^nd^ dose. Based on the lipid clearance dynamics of the oral fat tolerance test which shows peak absorptive activity at a 2–3‐h timepoint, we tested a 3‐h food withdrawal before drug administration to eliminate absorption‐related effects. This regimen ameliorated diarrhea‐like symptoms suggesting an eased intestinal burden. A consistent reduction in circulating FAs and RER in adipose‐specific *Dgat1/Dgat2* double knockout indicates toward adipose‐centric phenotype (Chandramohan *et al*, 2023). Moreover, the inhibition of intestinal lipid absorption would lead to reduced circulating FA levels that would intuitively lead to higher RER due to increasing carbohydrate oxidation as shown in the re‐feeding experiment after D1 + 2i (Fig 2B, lower panel). This indicates that the reduction in the circulating FA is plausibly a consequence of FAO in adipose tissue and to some extent in other organs (Fig 2D and E).

The second issue is about the source of FA that is oxidized upon D1 + 2i. Adipose tissue depots show a constant basal lipolysis and re‐esterification cycle, known as futile lipid cycling. The rate of lipolysis as well as of re‐esterification is increased in fasting or cold exposure. DGAT1/2 are the best‐known enzyme with diacylglycerol acyl‐transferase activity, the terminal and only committed step in triglyceride synthesis. They use acyl‐CoA to transfer the third FA onto the DAG molecule. Inhibiting the activity of both enzymes would lead to excess acyl‐CoA which is plausibly diverted to mitochondria for FAO (Sharma *et al*, 2023).

Thirdly, the signaling pathway that mediates these effects needs further attention. Combined inhibition of DGAT1/2 leads to AMP‐mediated activation of AMPK that facilitates the FAO effect of D1 + 2i (Sharma *et al*, 2023). Moreover, acyl‐CoA is known to directly activate AMPK (Pinkosky *et al*, 2020). Therefore, AMPK seems to be a key mediator of the D1 + 2i effect on FAO. Consistently, as shown in this report (Fig 2C), simultaneous activation of AMPK with D1 + 2i shows a synergistic effect on FAO in adipocytes.

Overall, these findings suggest a possible therapeutic potential of DGAT inhibitors in different combinations. Further studies and trials are needed to examine the translatability of the findings.

## Limitations of the study

The findings presented in this correspondence report the effect of a single dose of DGAT inhibitors. Further studies are needed to assess the effects of chronic D1 + 2i treatment. Furthermore, although AMPK appears to be a potential mediator of these effects, the mechanistic aspect is still not clear at this point. Similarly, a more detailed study of the intestinal versus adipose‐specific effects of the D1 + 2i is needed.

## Materials and Methods

Chemical inhibitors and activators were purchased from Sigma Aldrich, Merck. All animal experiments were approved by the Zurich cantonal animal ethics committee (approval number 148/2020). Six‐week‐old male C57BL/6N mice were purchased from Charles River laboratories. After a 2–3‐week acclimatization to our facility conditions, experiments were performed when the mice were 8–10 weeks old. The mice were housed in individually ventilated cages under an inverted light cycle. For high‐fat diet‐fed mice, 4‐week‐old mice were ordered from the above‐mentioned vendor. The HFD was started when the mice were 6 weeks old. Experiments were performed after ~12 weeks of HFD. D1 + 2i was administered as described before (Takemoto *et al*, 2020). Blood samples were collected in EDTA‐coated collection tubes from the tail vein and plasma was separated by centrifugation. Blood glucose was measured by handheld glucometer (Accu‐Check Aviva, Roche). Plasma insulin was measured using the ultrasensitive ELISA kit (Crystal Chem, 90080). Plasma FAs, glycerol, and seahorse extracellular flux analysis were performed as described earlier (Sharma *et al*, 2023). Indirect calorimetry was performed on the TSE Phenomaster system. The *in vitro* FAO assay was performed using tritiated palmitate as the tracer. Briefly, mouse brown‐adipose tissue‐derived immortalized pre‐adipocytes (iBA cell, Kind gift from Dr Ronald Kahn, Joslin Diabetes Center) were differentiated on collagen‐coated P10 cell culture dishes as described previously (Sharma *et al*, 2023). On day 5 of differentiation, adipocytes were replated on collagen‐coated 24‐well cell culture plates. On the day of the experiment (day 7 of differentiation), cells were fasted for 2 h in DMEM containing 0.2% BSA. Next, cells were treated with the tritiated palmitate (Perkin Elmer). The corresponding pharmacological agents were mixed in the tracer mix (DGAT1 or 2 inhibitors were used at 2 μM working concentration, AMPK activator concentration was 5 μM, while etomoxir was used at 100 μM working concentration). After 6‐h tracer incubation (0.5 μCi/ml; 300 μl/well with corresponding pharmacological agents) in minimal DMEM containing 0.2% BSA, 200 μl supernatant was collected and incubated for 30 min with activated charcoal. After 15 min centrifugation, 300 μl supernatant was mixed with 2.7 ml of scintillation mix, incubated overnight, and read the radioactivity. For the intracellular tracer accumulation measurement, cells were washed 5× with PBS containing 0.1% tween‐20. The cells were lysed in 150 μl 0.4 M NaOH and radioactivity was measured in the whole fraction.

Details of *in vitro* oxygen consumption rate (OCR) measurement in adipocytes and HepG2 cells are provided in the legend section. Adipocytes were differentiated as described above. On day 5, adipocytes were replated on collagen coated XFe96 cell culture plates, and the OCR measurement was performed on day 7. The inhibitor treatment (2 μM) was done for 1 h before the OCR measurement as described previously (Sharma *et al*, 2023). Human hepatoma HepG2 cells (ATCC, HB‐8065) were used directly as described in the figure legends. Cultured cells in the lab are tested for mycoplasma at regular intervals using LookOut^®^ Mycoplasma PCR‐Detections‐Kit (Merck, MP0035‐1KT).

No blinding or sample size estimate was done during the experiment or the analysis. No formal randomization was performed, although cage mates and littermates were evenly distributed across treatment groups to avoid the cage effect. No animals or samples were excluded from analysis. The details of the test used and the number of biological replicates in each group are mentioned in the figure legend. The *P* values of each data point are mentioned in the figures.

## Author contributions

**Anand Kumar Sharma:** Conceptualization; data curation; formal analysis; investigation; visualization; methodology; writing – original draft; project administration; writing – review and editing. **Christian Wolfrum:** Formal analysis; supervision; funding acquisition; project administration; writing – review and editing.

## Supporting information



Source Data for Figure 1

Source Data for Figure 2

## Data Availability

This study includes no data deposited in external repositories.
