# Supplementary material for: DGAT inhibition at the post‐absorptive phase reduces plasma FA by increasing FA oxidation
Source: EMBO Mol Med. 2023 Oct 4;15(11):e18209. doi: 10.15252/emmm.202318209 (PMC10630880; doi:10.15252/emmm.202318209)
Supplement: Supplementary file 3 [file EMMM-15-e18209-s002.pdf]

EMBO Molecular Medicine

DGAT inhibition at post-absorptive phase reduces plasma fatty acids by increasing FA oxidation

Anand Sharma and Christian Wolfrum
DOI: 10.15252/emmm.202318209

Corresponding author: Anand Sharma (anand.sharma@hest.ethz.ch) , Christian Wolfrum (christian-wolfrum@ethz.ch)

Review Timeline:

Submission Date:	23rd Jun 23
Editorial Decision:	12th Jul 23
Appeal Received:	14th Jul 23
Editorial Decision:	14th Jul 23
Revision Received:	27th Jul 23
Editorial Decision:	4th Sep 23
Revision Received:	12th Sep 23
Accepted:	18th Sep 23

Editor: Lise Roth

Transaction Report:

(Note: With the exception of the correction of typographical or spelling errors that could be a source of ambiguity, letters and reports are not edited. Depending on transfer agreements, referee reports obtained elsewhere may or may not be included in this compilation. Referee reports are anonymous unless the Referee chooses to sign their reports.)

12th Jul 2023

Dear Dr. Sharma,

Thank you for submitting your manuscript to EMBO Molecular Medicine. We have now heard back from the 2 referees who agreed to evaluate your manuscript.

As you will see from the reports below, the referees provide rather similar recommendations. They both recognize the potential clinical interest of your findings, however, they also agree that the results remain preliminary without in vivo validation, and that the mechanistic insight is not sufficient at this point to support the conclusions.

Given these considerations, I am afraid I see little choice but to return the manuscript to you with the decision that we cannot offer to publish it.

However, given the interest of your findings, we would be happy to consider a new manuscript on the topic if you were to address these points (as Report or Research Article).

I thank you again for your interest in EMBO Molecular Medicine and I am sorry that I could not bring better news. I hope that the referees' comments are helpful in your continued work in this area.

Yours sincerely,

Lise Roth

Lise Roth, PhD
Senior Editor
EMBO Molecular Medicine

***** Reviewer's comments *****

Referee #1 (Remarks for Author):

In this short correspondence, Sharma and Wolfrum present a novel use of DGAT 1 and 2 inhibitors as anti-obesity agents that reduce plasma levels of free fatty acids via increasing fatty acid oxidation in the adipose tissue. The data shown are interesting and might be of clinical importance in the future especially if gastrointestinal side effects of DGAT1i are avoided when following the protocol proposed by the authors. However, several concerns should be addressed to support the claims of the authors. Honestly, I am not sure if the experiments required to support the claims of the authors can be presented in a short format of "Correspondence". However, the concept is definitely worth bringing forward.

a) It is claimed that the observed effect of DGAT1/2 inhibition was absorption-independent but it was not really verified. Why was the timing 3 h post food withdrawal chosen as free from absorption? For this, either the activity of intestinal DGAT1/2 or lipid transport across the intestine should be determined at given conditions of drug administration. Moreover, DGAT1/2 will block the synthesis of triglycerides from the existing free fatty acids (monoglycerides) - so where do these fatty acids come from?

b) The authors claim that their results might bring back DGAT1/2 inhibitors into clinical focus. However, in their study, they used only a single dose of the inhibitors. What happens when multiple doses of the inhibitors are used? Is the effect of these compounds still absorption-independent?

c) It is not described which type of adipose tissue is studied, both in vitro and in vivo and this will have systemic consequences - does DGAT1/2 inhibition increase fatty acid utilization in white adipose and blocks the formation of fat depots or boosts the function of brown adipose? What about FAO in other organs?

d) Although the impact of DGAT1/2 inhibition on fatty acid metabolism in adipocytes in vitro is clear, such a link in vivo is not proven. The authors should be either more careful when postulating the impact of DGAT1/2 blockage on FAO specifically in adipose or determine FAO in DGAT1/2 adipocyte-specific knockout animals upon the treatment with inhibitors.

e) I don't see the point to include in this version of the manuscript Figure 1h. First of all, why the authors studied the expression of the genes related to lipid metabolism in the liver? They do not show any functional assays (like FAO) done in this organ. Second, in contrast to this what is stated in the manuscript, some significant changes in the expression of genes related to lipid metabolism are presented in Figure 1h (at least 3 genes). Without further molecular and functional analyses, these data are simply impossible to interpret.

f) What is the purpose of using DGAT1/2 inhibitors in mice exposed to the cold? I think these experiments are irrelevant in the context of re-proposing DGAT1/2 inhibitors as a therapy against obesity. At least in the current form of the manuscript, the link is not explained.

Minor:
a) In the sentence: "In humans, DGAT1 seems to be indispensable for dietary lipid absorption (...) " a supporting reference is missing.
b) In the same sentence: "(...) while in mice DGAT2 efficiently compensates for the loss of DGAT1 activity (Chitraju et al., 2019) " - this might suggest compensation for intestinal DGAT1, which is misleading since Chitraju et al. describes such an overlap in the adipose tissue.
c) "Consequently, although DGAT1 loss of function in humans causes severe diarrhea-like symptoms, Dgat1 (...) " - the gene name writing should be unified.
d) Figure 1 g description: "Etomoxir slightly decreased the FAP (...) "- typo
e) Curves on graphs in Figure 1 b- d are not described. Similarly, the bars in Figure 1 g, h.
f) The authors should indicate the number of biological replicates used for each experiment.

Referee #2 (Novelty/Model system Comments for Author):

The study relies solely on the use of chemical inhibitors of DGAT and activators of AMPK to draw the conclusion, which are no specific and inconclusive. More specific approaches such as DGAT1/2 KO mice and ectopic expression of constitutively active AMPK, should be used.

Referee #2 (Remarks for Author):

In this short communication, the authors provided a single figure showing that pharmacological inhibition of DGAT1/2 at post-operative phase reduces plasma FFAs by increasing FAO. However, the findings are rather preliminary with little mechanistic explanation.
1) Although the authors observed increased FAO by DGAT1/2 inhibitor in brown adipocytes (panel g), it does not decreased plasma FFAs in mice is attributed to increased FAO. There are many other factors which can modulate FFA levels such as lipolysis, lipogenesis.
2) There is no mechanistic explanation at all on how pharmacological inhibition of DAGT1/2 can increase FAO.

As a service to authors, EMBO provides authors with the possibility to transfer a manuscript that one journal cannot offer to publish to another EMBO publication. The full manuscript and if applicable, reviewers reports are automatically sent to the receiving journal to allow for fast handling and a prompt decision on your manuscript. For more details of this service, and to transfer your manuscript to another EMBO title please click on Link Not Available

Please do not share this URL as it will give anyone who clicks it access to your account.

We are thankful to both the reviewers for very insightful comments. Incorporating information to address these points will definitely improve the manuscript. We would like to highlight that we were over-conscious of exceeding the word limit which led some aspects remain incompletely discussed and explained. As you would see from our response to specific comments, majority of these points can be addressed by adding existing information and provide focused arguments in the manuscript text. We hope that with our response, the reviewers are persuaded that the data presented here are sufficient to justify as a Correspondence.

Referee #1 (Remarks for Author):

In this short correspondence, Sharma and Wolfrum present a novel use of DGAT 1 and 2 inhibitors as anti-obesity agents that reduce plasma levels of free fatty acids via increasing fatty acid oxidation in the adipose tissue. The data shown are interesting and might be of clinical importance in the future especially if gastrointestinal side effects of DGAT1i are avoided when following the protocol proposed by the authors. However, several concerns should be addressed to support the claims of the authors. Honestly, I am not sure if the experiments required to support the claims of the authors can be presented in a short format of "Correspondence". However, the concept is definitely worth bringing forward.

We thank the reviewer for very positive feedback.

a) It is claimed that the observed effect of DGAT1/2 inhibition was absorption-independent but it was not really verified. Why was the timing 3 h post food withdrawal chosen as free from absorption? For this, either the activity of intestinal DGAT1/2 or lipid transport across the intestine should be determined at given conditions of drug administration.

We agree with the reviewer that this is a crucial point to be addressed. Our conclusions are based on the following points:

A) The 3 h post food withdrawal was selected based on our experience with the Fat Tolerance Test (oral gavage of olive oil and monitoring the clearance). In our experience, the peak of the plasma fat reaches in 2 hours and the majority is cleared in the 4h. Since we administered the DGATi at 3h post food withdrawal, the absorption and action of DGATi coincide with the time point at which most of the gastric fat is cleared. Moreover, we have done one batch of experiments with 5h food withdrawal; overall the results were consistent but towards the later time points the fasting effect comes in, therefore we selected 3h food withdrawal which clears gastric fat but doesn't invoke a fasting response yet.

B) Chitraju et al recently characterized adipose-specific Dgat1/2 double knockout and found a similar effect i.e., reduced circulating FA combined with reduced RER. This indicates that adipose-specific loss of DGAT1/2 loss of activity is the key driver of this phenotype (Chitraju et al., 2023, eLife). In this report, authors saw an increased Ucp1 expression in the non-brown adipose depots and speculated that the browning could be a driver of this phenotype. However, our ongoing experiments suggest that Ucp1 upregulation may not be responsible for this phenotype but instead be a consequence (please see next point). Mechanistically, AMPK activation by AMP and Acyl-CoA is a much more likely possibility and we have included that in the revised manuscript.

c) [...] The figure on the left is from Chitraju et al., (2023) where HFD fed the Dgat1/2 double knockout shows a drastic reduction in the RER. [...]

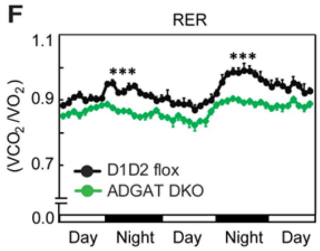

Figure (and related text above) for referees removed.

D) The acute and visually discernible gastrointestinal side-effect of combined DGATi in mice (and a corresponding effect of DGAT1i in humans) is watery diarrhoea, especially if the mice are on HFD. We also observed a diarrhoea-like symptom in non-fasted mice while this symptom was mitigated when the drug was administered after 3h of food withdrawal. We will add this information in the revised manuscript.

E) If the phenotype was due to defective lipid absorption, one would expect a higher RER due to increased carbohydrate oxidation which is not the case here.

Moreover, DGAT1/2 will block the synthesis of triglycerides from the existing free fatty acids (monoglycerides) - so where do these fatty acids come from?

As the author rightly pointed out, the DGATs block the re-esterification. The origin of these intracellular lipid is from basal lipolysis which is always happening in the adipose tissue even in the fed state (and this constitutes a futile lipid cycle). After DGAT inhibition, the fatty acids that were to be re-esterified are now free to be used for FAO. In this regard, AMPK seems to play an important role of facilitate and sustain the FAO. We have now edited the manuscript text to clarify this issue.

b) The authors claim that their results might bring back DGAT1/2 inhibitors into clinical focus. However, in their study, they used only a single dose of the inhibitors. What happens when multiple doses of the inhibitors are used? Is the effect of these compounds still absorption-independent?
As evident from the adipose-specific Dgat1/2 knockout data presented above, the results are most likely to be chronic. However, at this point, we have no data on chronic pharmacological treatment. However, we believe that this short report is more like a proof of concept that needs further exploration, especially in a pilot clinical trial.

c) It is not described which type of adipose tissue is studied, both in vitro and in vivo and this will have systemic consequences - does DGAT1/2 inhibition increase fatty acid utilization in white adipose and blocks the formation of fat depots or boosts the function of brown adipose? What about FAO in other organs?
In our recent manuscript (which is foundational work for this report, Sharma et al., 2023, Mol Metab) we have shown that in vitro DGATi increases FAO in both white and brown adipose tissue. A hallmark of this effect was a large increased OCR. We also performed a seahorse assay with HepG2 cells (unpublished data). We found a similar trend, but the magnitude was much smaller. This is likely because the DGAT activity is highest in the adipose tissue. We can add this data in the revised manuscript.

Effect of DGATi on the OCR in HepG2 cells (left) or brown adipocytes in vitro (right).

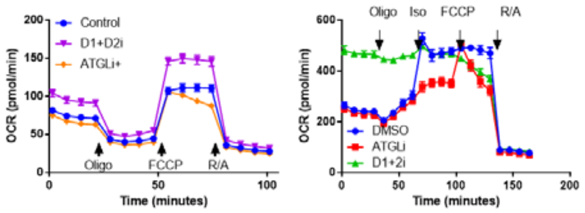

d) Although the impact of DGAT1/2 inhibition on fatty acid metabolism in adipocytes in vitro is clear, such a link in vivo is not proven. The authors should be either more careful when postulating the impact of DGAT1/2 blockage on FAO specifically in adipose or determine FAO in DGAT1/2 adipocyte-specific knockout animals upon the treatment with inhibitors.

In the previous version of the manuscript, we showed a significant drop in the RER which was coincident with the reduced plasma fatty acid levels. That suggested that in vivo, FAs are oxidized more in the presence of DGATi. With the description of the adipose-specific knockout mice, I hope that the reviewer is convinced about the specificity. We will briefly clarify this aspect in the revised manuscript.

e) I don't see the point to include in this version of the manuscript Figure 1h. First of all, why the authors studied the expression of the genes related to lipid metabolism in the liver? They do not show any functional assays (like FAO) done in this organ. Second, in contrast to this what is stated in the manuscript, some significant changes in the expression of genes related to lipid metabolism are presented in Figure 1h (at least 3 genes). Without further molecular and functional analyses, these data are simply impossible to interpret.

As suggested by the reviewer, we will remove this data point.

f) What is the purpose of using DGAT1/2 inhibitors in mice exposed to the cold? I think these experiments are irrelevant in the context of re-proposing DGAT1/2 inhibitors as a therapy against obesity. At least in the current form of the manuscript, the link is not explained.
The main objective for the comparison to RT vs cold was to see if FA availability is the major driver of the phenotype. Also, it is known that cold exposure also leads to a higher rate of re-esterification by DGATs, a phenomenon known as futile cycling. It was important to differentiate the effect of DGATi at resting and at cold-stimulated state. Consistently, in the cold-exposed mice, the reduction in the RER was more pronounced. We will clarify this point in the revised manuscript.

Minor:
a) In the sentence: "In humans, DGAT1 seems to be indispensable for dietary lipid absorption (...) " a supporting reference is missing.
b) In the same sentence: "(...) while in mice DGAT2 efficiently compensates for the loss of DGAT1 activity (Chitraju et al., 2019) " - this might suggest compensation for intestinal DGAT1, which is misleading since Chitraju et al. describes such an overlap in the adipose tissue.
c) "Consequently, although DGAT1 loss of function in humans causes severe diarrhea-like symptoms, Dgat1 (...) " - the gene name writing should be unified.
d) Figure 1 g description: "Etomoxir slightly decreased the FAP (...) "- typo
e) Curves on graphs in Figure 1 b- d are not described. Similarly, the bars in Figure 1 g, h.
f) The authors should indicate the number of biological replicates used for each experiment.
We thank the reviewer for these inputs. These points will be taken care of in the revised manuscript.

Referee #2 (Novelty/Model system Comments for Author):

The study relies solely on the use of chemical inhibitors of DGAT and activators of AMPK to draw the conclusion, which are no specific and inconclusive. More specific approaches such as DGAT1/2 KO mice and ectopic expression of constitutively active AMPK, should be used.

We thank the reviewer for the very helpful suggestion. Although we are unable to provide data on the constitutive active AMPK mouse model, we believe that our discussion on the adipose-specific Dgat1/2 double knockouts substantiates our conclusions. Moreover, we now provide another reference that very strongly suggests that AMPK could be the key driver.

Moreover, very recently, we thoroughly validated the role of AMPK signalling in mediating the role of DGATi in adipocytes (Sharma et al., 2023, Mol Metab). We will include some more information in the revised text to highlight this aspect.

Pinkosky et al. showed that AMPK can also be directly activated by activated fatty acids (Acyl-CoA). Since after the blockage of DGATs, there will be an excess pool of Acyl-CoA, this provides a link between the two pathways. We will include this information and a short schematic for a better representation of this mechanism.

*Pinkosky, S.L., Scott, J.W., Desjardins, E.M. et al. Long-chain fatty acyl-CoA esters regulate metabolism via allosteric control of AMPK β1 isoforms. Nat Metab 2, 873–881 (2020). https://doi.org/10.1038/s42255-020-0245-2

Referee #2 (Remarks for Author):

In this short communication, the authors provided a single figure showing that pharmacological inhibition of DGAT1/2 at post-operative phase reduces plasma FFAs by increasing FAO. However, the findings are rather preliminary with little mechanistic explanation.
1) Although the authors observed increased FAO by DGAT1/2 inhibitor in brown adipocytes (panel g), it does not decreased plasma FFAs in mice is attributed to increased FAO. There are many other factors which can modulate FFA levels such as lipolysis, lipogenesis.
2) There is no mechanistic explanation at all on how pharmacological inhibition of DAGT1/2 can increase FAO.

We agree with the reviewer that we did not present much of the mechanistic data here. This is also partly because we recently established the role of AMPK signaling in mediating the effect of DGATi in adipocytes. We will provide a better discussion on this in the revised manuscript.

1) We agree that a reduced plasma FA could originate from different mechanisms. However, our in vivo indirect calorimetry data clearly indicates reduced RER which is because of increased FAO at the systemic level. Therefore, the two data together suggest FAO. Moreover, our better description of the phenotype of the adipose-specific Dgat1/2 knockout mice unequivocally proves the adipose-centric FAO in vivo.
2) We will edit the text to better explain the mechanistic activation of AMPK by DGATi as we have published this aspect very recently. In addition, we will add a small schematic for a better representation (Fig below). Inhibition of DGATs increases Acyl CoA levels and AMP levels that may activate and facilitate the AMPK-mediated FAO.

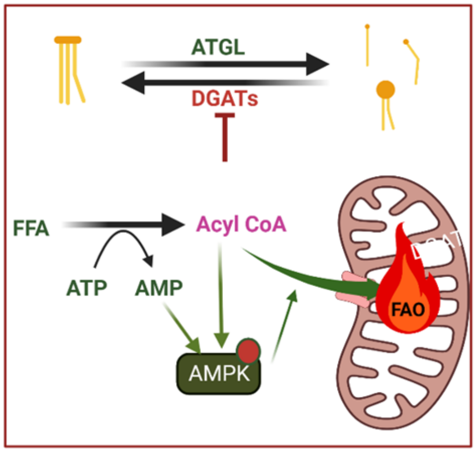
ATGL
DGATs
FFA
Acyl CoA
ATP
AMP
AMPK
FAO

14th Jul 2023

Dear Anand,

Thank you for your appeal asking us to reconsider our decision on your manuscript.

I have carefully read your letter and discussed it with my colleagues here. As mentioned in my initial decision letter, we agree with the referees that the concept developed in your manuscript is worth being spread to the community. Given the extent of the referees' concerns, we thought a longer article might allow you to address all the issues raised. However, from your letter I understand this might not be feasible, and we thus decided to reconsider our decision and invite revision of your manuscript as a "correspondence".
As you rightly noted, we are flexible in terms of formatting, and could accommodate longer text.

Please note however that a revised version should nevertheless address the main referees' concerns and that the data should convincingly support the conclusions. The manuscript will be subjected to re-review, and I cannot guarantee at this stage that the outcome will be favorable.

EMBO Molecular Medicine encourages a single round of revision only and therefore, acceptance or rejection of the manuscript will depend on the completeness of your responses included in the next, final version of the manuscript.

Please read below for important editorial formatting and consult our author's guidelines for proper formatting of your revised article for EMBO Molecular Medicine.

I look forward to seeing a revised form of your manuscript as soon as possible. Use this link to login to the manuscript system and submit your revision:

With kind regards,

Lise

Lise Roth, PhD
Senior Editor
EMBO Molecular Medicine

*****

When submitting your revised manuscript, please carefully review the instructions that follow below. We perform an initial quality control of all revised manuscripts before re-review; failure to include requested items will delay the evaluation of your revision.

We require:

1) A .docx formatted version of the manuscript text (including legends for main figures, EV figures and tables). Please make sure that the changes are highlighted to be clearly visible.

2) Individual production quality figure files as .eps, .tif, .jpg (one file per figure). For guidance, download the 'Figure Guide PDF': (https://www.embopress.org/page/journal/17574684/authorguide#figureformat).

3) A .docx formatted letter INCLUDING the reviewers' reports and your detailed point-by-point responses to their comments. As part of the EMBO Press transparent editorial process, the point-by-point response is part of the Review Process File (RPF), which will be published alongside your paper.

4) A complete author checklist, which you can download from our author guidelines (https://www.embopress.org/page/journal/17574684/authorguide#submissionofrevisions). Please insert information in the checklist that is also reflected in the manuscript. The completed author checklist will also be part of the RPF.

5) Please note that all corresponding authors are required to supply an ORCID ID for their name upon submission of a revised manuscript.

6) It is mandatory to include a 'Data Availability' section after the Materials and Methods. Before submitting your revision, primary

datasets produced in this study need to be deposited in an appropriate public database, and the accession numbers and database listed under 'Data Availability'. Please remember to provide a reviewer password if the datasets are not yet public (see https://www.embopress.org/page/journal/17574684/authorguide#dataavailability).

In case you have no data that requires deposition in a public database, please state so in this section. Note that the Data Availability Section is restricted to new primary data that are part of this study.

7) For data quantification: please specify the name of the statistical test used to generate error bars and P values, the number (n) of independent experiments (specify technical or biological replicates) underlying each data point and the test used to calculate p-values in each figure legend. The figure legends should contain a basic description of n, P and the test applied. Graphs must include a description of the bars and the error bars (s.d., s.e.m.). See also 'Figure Legend' guidelines: https://www.embopress.org/page/journal/17574684/authorguide#figureformat

8) At EMBO Press we ask authors to provide source data for the main manuscript figures. Our source data coordinator will contact you to discuss which figure panels we would need source data for and will also provide you with helpful tips on how to upload and organize the files.

9) Author contributions: You will be asked to provide CRediT (Contributor Role Taxonomy) terms in the submission system. These replace a narrative author contribution section in the manuscript.

10) A Conflict of Interest statement should be provided in the main text.

EMBO Molecular Medicine has a "scooping protection" policy, whereby similar findings that are published by others during review or revision are not a criterion for rejection. Should you decide to submit a revised version, I do ask that you get in touch after three months if you have not completed it, to update us on the status.

Please note: When submitting your revision you will be prompted to enter your funding and payment information. This will allow Wiley to send you a quote for the article processing charge (APC) in case of acceptance. This quote takes into account any reduction or fee waivers that you may be eligible for. Authors do not need to pay any fees before their manuscript is accepted and transferred to the publisher.

EMBO Press participates in many Publish and Read agreements that allow authors to publish Open Access with reduced/no publication charges. Check your eligibility: https://authorservices.wiley.com/author-resources/Journal-Authors/open-access/affiliation-policies-payments/index.html

We would like to thank both the reviewers for their insightful comments. Addressing these points has improved the manuscript. We would like to stress that we strictly adhered to the word limit, which led to some aspects remaining incompletely discussed and explained. As you can see from our response to specific comments, these points are now addressed by adding supporting data and focused arguments/explanations in the manuscript text. We hope that the revised version is acceptable as a Correspondence.

Referee #1 (Remarks for Author):

In this short correspondence, Sharma and Wolfrum present a novel use of DGAT 1 and 2 inhibitors as anti-obesity agents that reduce plasma levels of free fatty acids via increasing fatty acid oxidation in the adipose tissue. The data shown are interesting and might be of clinical importance in the future especially if gastrointestinal side effects of DGAT1i are avoided when following the protocol proposed by the authors. However, several concerns should be addressed to support the claims of the authors. Honestly, I am not sure if the experiments required to support the claims of the authors can be presented in a short format of "Correspondence". However, the concept is definitely worth bringing forward.

We thank the reviewer for very positive feedback. We agree that adding more mechanistic data would have been interesting, nevertheless, we believe that the revised manuscript is suitable and acceptable as a Correspondence.

a) It is claimed that the observed effect of DGAT1/2 inhibition was absorption-independent but it was not really verified. Why was the timing 3 h post food withdrawal chosen as free from absorption? For this, either the activity of intestinal DGAT1/2 or lipid transport across the intestine should be determined at given conditions of drug administration.

We agree with the reviewer that this is a crucial point to be addressed. We have added some data (Fig 2B, lower panel) and few other circumstantial evidence to prove the specificity of the phenotype.

A) If the phenotype was due to compromised lipid absorption, one would expect a higher RER due to increased carbohydrate oxidation which is not the case here. Consistently, when we performed a re-feeding experiment after D1+2i administration, we observed and increased RER, thus demonstrating the reduction in RER is not related to absorption and reduction in the plasma FAs is most likely a consequence of FAO.

B) The 3 h post food withdrawal was selected based on our experience with the Fat Tolerance Test (oral gavage of olive oil and monitoring the clearance). In our experience, the peak of the plasma fat reaches in 2 hours and the majority is cleared in the 4h. Since we administered the DGATi at 3h post food withdrawal, the absorption and action of DGATi coincide with the time point at which most of the intestinal fat is cleared. Moreover, we have done one batch of experiments with 5h food withdrawal; overall the results were consistent but towards the later time points the fasting effect comes in, therefore we selected 3h food withdrawal which clears gastric fat but doesn’t invoke a fasting response yet.

C) Chitraju et al recently characterized adipose-specific *Dgat1/2* double knockout and found a similar effect i.e., reduced circulating FA combined with reduced RER. This indicates that adipose-specific loss of DGAT1/2 loss of activity is the major driver of this phenotype (Chitraju et al., 2023, eLife).

D) The acute and visually discernible gastrointestinal side-effect of combined DGATi in HFD-fed mice (and a corresponding effect of DGAT1i in humans) is watery diarrhoea. We observed a diarrhoea-like symptom in non-fasted mice while this symptom was mitigated when the drug was administered after 3h of food withdrawal.

Moreover, DGAT1/2 will block the synthesis of triglycerides from the existing free fatty acids (monoglycerides) - so where do these fatty acids come from?

As the reviewer rightly pointed out, DGATs block the re-esterification. The origin of these intracellular lipid is from basal lipolysis which is always happening in the adipose tissue even in the fed state (and this constitutes a futile lipid cycle). After DGAT inhibition, the fatty acids that were to be re-esterified are now free to be used for FAO. In this regard, AMPK seems to play an important role of facilitate and sustain the FAO. We have now edited the manuscript text to clarify this issue. Unfortunately, we cannot cite many of these excellent studies due to limited number of citations allowed in a Correspondence.

1. Rydén, M., Andersson, D. P., Bernard, S., Spalding, K. & Arner, P. Adipocyte triglyceride turnover and lipolysis in lean and overweight subjects. Journal of Lipid Research 54, 2909–2913 (2013).
2. Arner, P., Andersson, D. P., Bäckdahl, J., Dahlman, I. & Rydén, M. Weight Gain and Impaired Glucose Metabolism in Women Are Predicted by Inefficient Subcutaneous Fat Cell Lipolysis. Cell Metabolism 28, 45-54.e3 (2018).
3. Rydén, M. et al. Lipolysis defect in people with obesity who undergo metabolic surgery. Journal of Internal Medicine 292, 667–678 (2022).

b) The authors claim that their results might bring back DGAT1/2 inhibitors into clinical focus. However, in their study, they used only a single dose of the inhibitors. What happens when multiple doses of the inhibitors are used? Is the effect of these compounds still absorption-independent?
As evident from the adipose-specific *Dgat1/2* double knockout data discussed above, the results are most likely to be chronic. However, at this point, we have no data on chronic pharmacological treatment. In one set of experiment, we observed a three-day consecutive injection was still consistent. We believe that this short report is more like a proof of concept that needs further exploration, especially in a pilot clinical trial.

c) It is not described which type of adipose tissue is studied, both in vitro and in vivo and this will have systemic consequences - does DGAT1/2 inhibition increase fatty acid utilization in white adipose and blocks the formation of fat depots or boosts the function of brown adipose? What about FAO in other organs?
In our recent manuscript (which is foundational work for this report, Sharma et al., 2023, *Mol Metab*) we have shown that *in vitro* DGATi increases FAO in both white and brown adipose tissue. A hallmark of this effect was a large increased OCR. We also performed a seahorse assay with HepG2 cells. We found a similar trend, but the magnitude was much smaller. Therefore, adipose tissue seems primary effector also because the rate of basal lipolysis and DGAT activity is highest in the adipose tissue. We have added these data in the revised manuscript with appropriate discussion (Fig 2D).

d) Although the impact of DGAT1/2 inhibition on fatty acid metabolism in adipocytes in vitro is clear, such a link in vivo is not proven. The authors should be either more careful when postulating the impact of DGAT1/2 blockage on FAO specifically in adipose or determine FAO in DGAT1/2 adipocyte-specific knockout animals upon the treatment with inhibitors.

We agree that other organs may also be involved in FAO oxidation. We have modified our statement to make this distinction as suggested.

We would like to highlight a recent study by Chitraju et al., (eLife, 2023) where adipose-specific knockout of Dgat1/2 led to reduced RER, indicating that the adipose tissue may be the major effector of this phenotype.

e) I don't see the point to include in this version of the manuscript Figure 1h. First of all, why the authors studied the expression of the genes related to lipid metabolism in the liver? They do not show any functional assays (like FAO) done in this organ. Second, in contrast to this what is stated in the manuscript, some significant changes in the expression of genes related to lipid metabolism are

presented in Figure 1h (at least 3 genes). Without further molecular and functional analyses, these data are simply impossible to interpret.

Thanks, you for this suggestion. We have now removed these data.

f) What is the purpose of using DGAT1/2 inhibitors in mice exposed to the cold? I think these experiments are irrelevant in the context of re-proposing DGAT1/2 inhibitors as a therapy against obesity. At least in the current form of the manuscript, the link is not explained.
The main objective for the comparison to RT vs cold was to see if FA availability is the major driver of the phenotype. It is known that cold exposure also leads to a higher rate of lipolysis and also re-esterification by DGATs, a phenomenon known as futile cycling. It was important to differentiate the effect of DGATi at resting and at cold-stimulated state. Consistently, in the cold-exposed mice, the reduction in the RER was more pronounced. We have clarified this point in the revised manuscript.

Minor:
a) In the sentence: "In humans, DGAT1 seems to be indispensable for dietary lipid absorption (...) " a supporting reference is missing.
b) In the same sentence: "(...) while in mice DGAT2 efficiently compensates for the loss of DGAT1 activity (Chitraju et al., 2019) " - this might suggest compensation for intestinal DGAT1, which is misleading since Chitraju et al. describes such an overlap in the adipose tissue.
c) "Consequently, although DGAT1 loss of function in humans causes severe diarrhea-like symptoms, Dgat1 (...) " - the gene name writing should be unified.
d) Figure 1 g description: "Etomoxir slightly decreased the FAP (...) "- typo
e) Curves on graphs in Figure 1 b- d are not described. Similarly, the bars in Figure 1 g, h.
f) The authors should indicate the number of biological replicates used for each experiment.
We thank the reviewer for these inputs. These points are taken care of in the revised manuscript.

Referee #2 (Novelty/Model system Comments for Author):

The study relies solely on the use of chemical inhibitors of DGAT and activators of AMPK to draw the conclusion, which are no specific and inconclusive. More specific approaches such as DGAT1/2 KO mice and ectopic expression of constitutively active AMPK, should be used.

We thank the reviewer for his/her helpful suggestions. Although we are unable to provide data on the constitutive active AMPK mouse model, we believe that our discussion on the adipose-specific Dgat1/2 double knockouts substantiates our conclusions. Moreover, we now provide another reference that very strongly suggests that AMPK could be the key driver.

Recently, we thoroughly validated the role of AMPK in mediating the role of DGATi in adipocytes (Sharma et al., 2023, *Mol Metab*). We will include some more information in the revised text to highlight this aspect. Similarly, Pinkosky et al. showed that AMPK is directly activated by Acyl-CoA. Since D1+2i will lead to excess Acyl-CoA, activation of AMPK by AMP as well as by Acyl-CoA is the mechanistic aspect that links the two pathways. We have included a schematic for a better representation of this mechanism (Fig 2E).

*Pinkosky, S.L., Scott, J.W., Desjardins, E.M. et al. Long-chain fatty acyl-CoA esters regulate metabolism via allosteric control of AMPK β1 isoforms. Nat Metab 2, 873–881 (2020). https://doi.org/10.1038/s42255-020-0245-2

Referee #2 (Remarks for Author):

In this short communication, the authors provided a single figure showing that pharmacological

inhibition of DGAT1/2 at post-operative phase reduces plasma FFAs by increasing FAO. However, the findings are rather preliminary with little mechanistic explanation.
1) Although the authors observed increased FAO by DGAT1/2 inhibitor in brown adipocytes (panel g), it does not decreased plasma FFAs in mice is attributed to increased FAO. There are many other factors which can modulate FFA levels such as lipolysis, lipogenesis.
2) There is no mechanistic explanation at all on how pharmacological inhibition of DAGT1/2 can increase FAO.

We agree with the reviewer that we did not present much of the mechanistic data here. This is also partly because we recently established the role of AMPK signaling in mediating the effect of DGATi in adipocytes. We now provide a better discussion on this in the revised manuscript.

1) We agree that a reduced plasma FA could originate from different mechanisms. However, our *in vivo* indirect calorimetry data clearly demonstrate a reduced RER which is because of increased FAO at the systemic level. Therefore, two data together suggest FAO. Moreover, our better description of the phenotype of the adipose-specific *Dgat1/2* knockout mice unequivocally proves the adipose-centric FAO *in vivo*.
2) We have edited the text to better explain the mechanistic activation of AMPK by D1+2i. In addition, we added a schematic for a better representation (Fig 2E). Inhibition of DGATs increases Acyl-CoA levels and AMP levels that plausibly activate and facilitate the AMPK-mediated FAO.

4th Sep 2023

Dear Dr. Sharma,

Thank you for the submission of your revised manuscript to EMBO Molecular Medicine, and please accept my apologies for the delay in getting back to you in this busy time of the year. We have now received the reports from the 2 referees who re-reviewed your manuscript. As you will see, they still raise the following concerns on your work:
1/ single dosage of DGAT inhibitor
2/ lack of mechanistic insight
However, they also mention that "the possible medical impact is high" (referee #1) and that "[the study] might be acceptable as a short communication. However, the limitation of this study should be discussed." (referee #2).
Having discussed your manuscript a second time with my colleagues, and taking into account the practical limitations discussed previously, we agree that the article is worth being published as a correspondence with the results at hand. However, we would like you to clearly state the limitations of the work in your manuscript, and overstatements should be avoided.

Furthermore, please also address the following editorial points:

1/ Main manuscript file:
- Please address the queries from our data editors in the related Data Edited manuscript file. Accept the previous changes, and only keep in track changes mode any new modification.
- Please provide a more detailed "Methods" section, and in particular provide information on mouse origin, gender, age, housing and husbandry conditions. Please also provide information on statistics (see checklist).
- Data Availability: It is mandatory to include a 'Data Availability' section after the Materials and Methods and before the Acknowledgements. Primary datasets produced in this study need to be deposited in an appropriate public database, and the accession numbers and database listed under 'Data Availability'. In case you have no data that requires deposition in a public database, please state so in this section ("This study includes no data deposited in external repositories").
- Please rename to the conflict of interest to "Disclosure statement and competing interests". We updated our journal's competing interests policy and request authors to consider both actual and perceived competing interests. Please review the policy https://www.embopress.org/competing-interests and update your competing interests if necessary.
- Please update the References format to alphabetical, with 10 names listed before et al.

2/ Figures:
- Please provide exact p values, not a range, including for non-significant (ns) p values.
- There is a reference to a Fig. 1G in the text, please correct.

3/ Please provide a complete author checklist, which you can download from our author guidelines (https://www.embopress.org/page/journal/17574684/authorguide#submissionofrevisions). Please insert information in the checklist that is also reflected in the manuscript. The completed author checklist will also be part of the RPF.

4/ As part of the EMBO Publications transparent editorial process initiative (see our Editorial at http://embomolmed.embopress.org/content/2/9/329), EMBO Molecular Medicine will publish online a Review Process File (RPF) to accompany accepted manuscripts.
This file will be published in conjunction with your paper and will include the anonymous referee reports, your point-by-point response and all pertinent correspondence relating to the manuscript. Let us know whether you agree with the publication of the RPF and as here, if you want to remove or not any figures from it prior to publication.
Please note that the Authors checklist will be published at the end of the RPF.

I look forward to receiving your revised manuscript.

Yours sincerely,

Lise Roth

Lise Roth, PhD
Senior Editor
EMBO Molecular Medicine

***** Reviewer's comments *****

Referee #1 (Novelty/Model system Comments for Author):

Additional experiments are required to justify the claims raised in the manuscript. At the current stage, the manuscript is not bringing sufficient advancements over previously published papers:
https://doi.org/10.1016/j.molmet.2023.101701 and https://doi.org/10.7554/eLife.88049.2
However, the possible medical impact is high, and generally, the methodology is adequate.

Referee #1 (Remarks for Author):

The authors have replied to most of the issues raised by the reviewer and overall, the manuscript is stronger in that form. Discussing point-by-point the limitations of the study balances the authors' claims and increases manuscript credibility. Also, including the data from re-feeding + DGAT1/2 inhibition supports the idea that applying the inhibitor 3 h after food withdrawal limits the contribution of intestinal DGATs. Still, that reasoning is deductive and indirect, while the statement could gain solid support by simply showing directly a minimized DGAT1/2 activity. That is also reflected in the nomenclature used, eg. 'intuitively', and 'plausibly', when speaking about the data which should be solid. The authors also claim that the presented data are kind of "proof of concept that needs further exploration, especially in a pilot clinical trial". At this stage, this is an overstatement. This claim would be justified if the authors would provide a piece of evidence that multiple doses (weeks of dosing) of an inhibitor are safe to use at least in rodents. Otherwise presented data are not bringing forward the concept of usage of DGAT inhibitors beyond cited by the authors publications:
https://doi.org/10.1016/j.molmet.2023.101701 and https://doi.org/10.7554/eLife.88049.2

Minor:

a) it should be clearly indicated in the main text that a single dose of inhibitors was tested in the study
b) the information about sex and age of animals are missing
c) in Figure 2 B please change x numeration to a 24 h clock format

Referee #2 (Novelty/Model system Comments for Author):

The conclusion was based solely on the use of a single dosage of DGAT inhibitor.

Referee #2 (Remarks for Author):

In this revised version, the authors did not provide any additional data to address my two major concerns: (1) decreased plasma FFAs in mice is attributed to increased FAO; (2)There is no mechanistic explanation at all on how pharmacological inhibition of DAGT1/2 can increase FAO. Instead, they provided further explanation to support their conclusion. This might be acceptable as a short communication. However, the limitation of this study should be discussed.

Response to reviewers

Referee #1 (Novelty/Model system Comments for Author):

Additional experiments are required to justify the claims raised in the manuscript. At the current stage, the manuscript is not bringing sufficient advancements over previously published papers: https://doi.org/10.1016/j.molmet.2023.101701 and https://doi.org/10.7554/eLife.88049.2
However, the possible medical impact is high, and generally, the methodology is adequate.

Referee #1 (Remarks for Author):
The authors have replied to most of the issues raised by the reviewer and overall, the manuscript is stronger in that form. Discussing point-by-point the limitations of the study balances the authors' claims and increases manuscript credibility. Also, including the data from re-feeding + DGAT1/2 inhibition supports the idea that applying the inhibitor 3 h after food withdrawal limits the contribution of intestinal DGATs. Still, that reasoning is deductive and indirect, while the statement could gain solid support by simply showing directly a minimized DGAT1/2 activity. That is also reflected in the nomenclature used, eg. 'intuitively', and 'plausibly', when speaking about the data which should be solid. The authors also claim that the presented data are kind of "proof of concept that needs further exploration, especially in a pilot clinical trial". At this stage, this is an overstatement. This claim would be justified if the authors would provide a piece of evidence that multiple doses (weeks of dosing) of an inhibitor are safe to use at least in rodents. Otherwise presented data are not bringing forward the concept of usage of DGAT inhibitors beyond cited by the authors publications:
https://doi.org/10.1016/j.molmet.2023.101701 and https://doi.org/10.7554/eLife.88049.2

Minor:
a) it should be clearly indicated in the main text that a single dose of inhibitors was tested in the study
b) the information about sex and age of animals are missing
c) in Figure 2 B please change x numeration to a 24 h clock format

We thank the reviewer for highlighting the relevance of our work and assessment of the methodology. We have taken care of the feasible points raised by the reviewer. We agree with the reviewer that additional future studies are needed to consolidate the concept described here. For clarity, we have now included a section in the manuscript to underline the limitations of this study.

We would like to emphasise that we understand the seriousness of overstatements as one quoted by the reviewer ("proof of concept that needs further exploration, especially in a pilot clinical trial"). We would like to mention that this statement was made in our response to reviewer's comment and not in the manuscript. In the manuscript text, as rightly pointed out by the reviewer, we have used non-definitive terms to distinguish findings from assumptions.

Regarding the x-axis of graphs in 2B, since the total duration of the measurement is less than 24h, we have plotted time on the x-axis with respect to the treatment point because it is easier to assess the duration of the effect of inhibitor treatment.

Regarding the cited papers, we acknowledge that the cited papers (one of them is from us) made the foundation of this work. However, neither of those studies relate to pharmacological aspects of DGAT inhibitors, which is the topic of this correspondence.

Referee #2 (Novelty/Model system Comments for Author):

The conclusion was based solely on the use of a single dosage of DGAT inhibitor.

Referee #2 (Remarks for Author):
In this revised version, the authors did not provide any additional data to address my two major concerns: (1) decreased plasma FFAs in mice is attributed to increased FAO; (2)There is no mechanistic explanation at all on how pharmacological inhibition of DAGT1/2 can increase FAO. Instead, they provided further explanation to support their conclusion. This might be acceptable as a short communication. However, the limitation of this study should be discussed.

We thank the review for very helpful comments in the previous round of the review and we appreciate the concerns. Therefore, we have included a section in the manuscript to underline the limitations of this study as suggested.

18th Sep 2023

Dear Dr. Sharma,

Thank you for bearing with the last minor editorial issues. I am pleased to inform you that your manuscript is accepted for publication and is now being sent to our publisher to be included in the next available issue of EMBO Molecular Medicine!

Please read below for additional IMPORTANT information regarding your article, its publication and the production process.

Congratulations on your interesting work,

With kind regards,

Lise Roth

Lise Roth, Ph.D
Senior Editor
EMBO Molecular Medicine

Follow us on Twitter @EmboMolMed
Sign up for eTOCs at embopress.org/alertsfeeds

*** *** *** IMPORTANT INFORMATION *** *** ***

SPEED OF PUBLICATION
The journal aims for rapid publication of papers, using using the advance online publication "Early View" to expedite the process: A properly copy-edited and formatted version will be published as "Early View" after the proofs have been corrected. Please help the Editors and publisher avoid delays by providing e-mail address(es), telephone and fax numbers at which author(s) can be contacted.

Should you be planning a Press Release on your article, please get in contact with embomolmed@wiley.com as early as possible, in order to coordinate publication and release dates.

LICENSE AND PAYMENT:

All articles published in EMBO Molecular Medicine are fully open access: immediately and freely available to read, download and share.

EMBO Molecular Medicine charges an article processing charge (APC) to cover the publication costs. You, as the corresponding author for this manuscript, should have already received a quote with the article processing fee separately. Please let us know in case this quote has not been received.

Once your article is at Wiley for editorial production you will receive an email from Wiley's Author Services system, which will ask you to log in and will present you with the publication license form for completion. Within the same system the publication fee can be paid by credit card, an invoice, pro forma invoice or purchase order can be requested.

Payment of the publication charge and the signed Open Access Agreement form must be received before the article can be published online.

PROOFS

You will receive the proofs by e-mail approximately 2 weeks after all relevant files have been sent o our Production Office. Please return them within 48 hours and if there should be any problems, please contact the production office at embopressproduction@wiley.com.

Please inform us if there is likely to be any difficulty in reaching you at the above address at that time. Failure to meet our deadlines may result in a delay of publication.

All further communications concerning your paper proofs should quote reference number EMM-2023-18209-V4 and be directed to the production office at embopressproduction@wiley.com.

EMBO Press Author Checklist

Corresponding Author Name: Anand Kumar Sharma	
Journal Submitted to: EMBO Molecular Medicine	
Manuscript Number: EMM-2023-18209	

USEFUL LINKS FOR COMPLETING THIS FORM
The EMBO Journal - Author Guidelines
EMBO Reports - Author Guidelines
Molecular Systems Biology - Author Guidelines
EMBO Molecular Medicine - Author Guidelines

Reporting Checklist for Life Science Articles (updated January
This checklist is adapted from Materials Design Analysis Reporting (MDAR) Checklist for Authors. MDAR establishes a minimum set of requirements in transparent reporting in the life sciences (see Statement of Task: 10.31222/osf.io/9sm4x). Please follow the journal's guidelines in preparing your
Please note that a copy of this checklist will be published alongside your article.

Abridged guidelines for figures
1. Data
The data shown in figures should satisfy the following conditions:
- the data were obtained and processed according to the field's best practice and are presented to reflect the results of the experiments in an accurate and unbiased manner.
- ideally, figure panels should include only measurements that are directly comparable to each other and obtained with the same assay.
- plots include clearly labeled error bars for independent experiments and sample sizes. Unless justified, error bars should not be shown for technical
- if n<5, the individual data points from each experiment should be plotted. Any statistical test employed should be justified.
- Source Data should be included to report the data underlying figures according to the guidelines set out in the authorship guidelines on Data

2. Captions
Each figure caption should contain the following information, for each panel where they are relevant:
- a specification of the experimental system investigated (eg cell line, species name).
- the assay(s) and method(s) used to carry out the reported observations and measurements.
- an explicit mention of the biological and chemical entity(ies) that are being measured.
- an explicit mention of the biological and chemical entity(ies) that are altered/varied/perturbed in a controlled manner.
- the exact sample size (n) for each experimental group/condition, given as a number, not a range;
- a description of the sample collection allowing the reader to understand whether the samples represent technical or biological replicates (including how many animals, litters, cultures, etc.).
- a statement of how many times the experiment shown was independently replicated in the laboratory.
- definitions of statistical methods and measures:
  - common tests, such as t-test (please specify whether paired vs. unpaired), simple χ^2 tests, Wilcoxon and Mann-Whitney tests, can be unambiguously identified by name only, but more complex techniques should be described in the methods section;
  - are tests one-sided or two-sided?
  - are there adjustments for multiple comparisons?
  - exact statistical test results, e.g., P values = x but not P values < x;
  - definition of 'center values' as median or average;
  - definition of error bars as s.d. or s.e.m.

Please complete ALL of the questions below.
Select "Not Applicable" only when the requested information is not relevant for your study.

Materials

Newly Created Materials	Information included in the manuscript?	In which section is the information available? (Reagents and Tools Table, Materials and Methods, Figures, Data Availability Section)
New materials and reagents need to be available; do any restrictions apply?	Not Applicable	

Antibodies	Information included in the manuscript?	In which section is the information available? (Reagents and Tools Table, Materials and Methods, Figures, Data Availability Section)
For **antibodies** provide the following information:<br>- Commercial antibodies: RRID (if possible) or supplier name, catalogue number and or/clone number<br>- Non-commercial: RRID or citation	Not Applicable	

DNA and RNA sequences	Information included in the manuscript?	In which section is the information available? (Reagents and Tools Table, Materials and Methods, Figures, Data Availability Section)
**Short novel DNA or RNA including primers, probes**: provide the sequences.	Not Applicable	

Cell materials	Information included in the manuscript?	In which section is the information available? (Reagents and Tools Table, Materials and Methods, Figures, Data Availability Section)
**Cell lines:** Provide species information, strain. Provide accession number in repository **OR** supplier name, catalog number, clone number, and/**OR** RRID.	Yes	Materials and Methods
**Primary cultures:** Provide species, strain, sex of origin, genetic modification status.	Not Applicable	
Report if the cell lines were recently **authenticated** (e.g., by STR profiling) and tested for mycoplasma contamination.	Yes	Materials and Methods

Experimental animals	Information included in the manuscript?	In which section is the information available? (Reagents and Tools Table, Materials and Methods, Figures, Data Availability Section)
**Laboratory animals or Model organisms:** Provide species, strain, sex, age, genetic modification status. Provide accession number in repository **OR** supplier name, catalog number, clone number, **OR** RRID.	Yes	Materials and Methods
**Animal observed in or captured from the field:** Provide species, sex, and age where possible**.**	Not Applicable	
Please detail **housing and husbandry conditions**.	Yes	Materials and Methods

Plants and microbes	Information included in the manuscript?	In which section is the information available? (Reagents and Tools Table, Materials and Methods, Figures, Data Availability Section)
**Plants:** provide species and strain, ecotype and cultivar where relevant, unique accession number if available, and source (including location for collected wild specimens).	Not Applicable	
**Microbes:** provide species and strain, unique accession number if available, and source.	Not Applicable	

Human research participants	Information included in the manuscript?	In which section is the information available? (Reagents and Tools Table, Materials and Methods, Figures, Data Availability Section)
If collected and within the bounds of privacy constraints report on age, sex and gender or ethnicity for all study participants.	Not Applicable	

Core facilities	Information included in the manuscript?	In which section is the information available? (Reagents and Tools Table, Materials and Methods, Figures, Data Availability Section)
If your work benefited from core facilities, was their service mentioned in the acknowledgments section?	Not Applicable	

Design

Study protocol	Information included in the manuscript?	In which section is the information available? (Reagents and Tools Table, Materials and Methods, Figures, Data Availability Section)
If study protocol has been **pre-registered, provide DOI in the manuscript**. For clinical trials, provide the trial registration number **OR** cite DOI.	Not Applicable	
Report the **clinical trial registration number** (at ClinicalTrials.gov or equivalent), where applicable.	Not Applicable	

Laboratory protocol	Information included in the manuscript?	In which section is the information available? (Reagents and Tools Table, Materials and Methods, Figures, Data Availability Section)
Provide DOI OR other citation details if **external detailed step-by-step protocols** are available.	Yes	For in vitro OCR measurement, we referred to doi: 10.1016/j.molmet.2023.101701.

Experimental study design and statistics	Information included in the manuscript?	In which section is the information available? (Reagents and Tools Table, Materials and Methods, Figures, Data Availability Section)
Include a statement about **sample size** estimate even if no statistical methods were used.	Yes	Materials and Methods
Were any steps taken to minimize the effects of subjective bias when allocating animals/samples to treatment (e.g. **randomization procedure**)? If yes, have they been described?	Yes	Materials and Methods
Include a statement about **blinding** even if no blinding was done.	Yes	Materials and Methods
Describe **inclusion/exclusion criteria** if samples or animals were excluded from the analysis. Were the criteria pre-established?<br><br>If sample or data points were omitted from analysis, report if this was due to attrition or intentional exclusion and provide justification.	Yes	Materials and Methods
For every figure, are **statistical tests** justified as appropriate? Do the data meet the assumptions of the tests (e.g., normal distribution)? Describe any methods used to assess it. Is there an estimate of variation within each group of data? Is the variance similar between the groups that are being statistically compared?	Yes	Materials and Methods, Figure legend

Sample definition and in-laboratory replication	Information included in the manuscript?	In which section is the information available? (Reagents and Tools Table, Materials and Methods, Figures, Data Availability Section)
In the figure legends: state number of times the experiment was **replicated** in laboratory.	Not Applicable	
In the figure legends: define whether data describe **technical or biological replicates**.	Yes	Figure

Ethics

Ethics	Information included in the manuscript?	In which section is the information available? (Reagents and Tools Table, Materials and Methods, Figures, Data Availability Section)
Studies involving **human participants**: State details of **authority granting ethics approval** (IRB or equivalent committee(s), provide reference number for approval.	Not Applicable	
Studies involving **human participants**: Include a statement confirming that **informed consent** was obtained from all subjects and that the experiments conformed to the principles set out in the WMA Declaration of Helsinki and the Department of Health and Human Services Belmont Report.	Not Applicable	
Studies involving **human participants: For** publication of **patient photos**, include a statement confirming that consent to publish was obtained.	Not Applicable	
Studies involving experimental **animals**: State details of **authority granting ethics approval** (IRB or equivalent committee(s), provide reference number for approval. Include a statement of compliance with ethical regulations.	Yes	Materials and Methods
Studies involving **specimen and field samples:** State if relevant **permits** obtained, provide details of authority approving study; if none were required, explain why.	Not Applicable	

Dual Use Research of Concern (DURC)	Information included in the manuscript?	In which section is the information available? (Reagents and Tools Table, Materials and Methods, Figures, Data Availability Section)
Could your study fall under dual use research restrictions? Please check biosecurity documents and list of **select agents and toxins** (CDC): https://www.selectagents.gov/sat/list.htm	Not Applicable	
If you used a select agent, is the security level of the lab appropriate and reported in the manuscript?	Not Applicable	
If a study is subject to dual use research of concern regulations, is the name of the **authority granting approval and reference number** for the regulatory approval provided in the manuscript?	Not Applicable	

Reporting

The MDAR framework recommends adoption of discipline-specific guidelines, established and endorsed through community initiatives. Journals have their own policy about requiring specific guidelines and recommendations to complement MDAR.

Adherence to community standards	Information included in the manuscript?	In which section is the information available? (Reagents and Tools Table, Materials and Methods, Figures, Data Availability Section)
State if relevant guidelines or checklists (e.g., **ICMJE, MIBBI, ARRIVE, PRISMA**) have been followed or provided.	Not Applicable	
For **tumor marker prognostic studies,** we recommend that you follow the **REMARK** reporting guidelines (see link list at top right). See author guidelines, under 'Reporting Guidelines'. Please confirm you have followed these guidelines.	Not Applicable	
For **phase II and III randomized controlled trials**, please refer to the **CONSORT** flow diagram (see link list at top right) and submit the CONSORT checklist (see link list at top right) with your submission. See author guidelines, under 'Reporting Guidelines'. Please confirm you have submitted this list.	Not Applicable	

Data Availability

Data availability	Information included in the manuscript?	In which section is the information available? (Reagents and Tools Table, Materials and Methods, Figures, Data Availability Section)
Have **primary datasets** been deposited according to the journal's guidelines (see 'Data Deposition' section) and the respective accession numbers provided in the Data Availability Section?	Not Applicable	
Were **human clinical and genomic datasets** deposited in a public access-controlled repository in accordance to ethical obligations to the patients and to the applicable consent agreement?	Not Applicable	
Are **computational models** that are central and integral to a study available without restrictions in a machine-readable form? Were the relevant accession numbers or links provided?	Not Applicable	
If publicly available data were reused, provide the respective **data citations in the reference list**.	Not Applicable	
